# Complete genome sequences of *Streptomyces* phages HazuAndZazu and Tubberson

**DOI:** 10.1128/mra.00361-25

**Published:** 2025-06-09

**Authors:** Nisha Shah, Phoenix S. Bryant, Meghna Chandrasekaran, Arya J. Chaudhari, Ramsha A. Chaudhary, Cole Cheng, Norah J. X. Jackson, Allison S. Kende, Agnes Koodaly, Rohan S. Kyasa, Iman Mahmood, Katherine J. McHarg, Isabella S. Naimi, Ayeoritse T. Tuedon, 2024 UMBC Phage Hunters, Eni Adesola, Zachary M. Smith, Steven M. Caruso

**Affiliations:** 1 Department of Biological Sciences, University of Maryland Baltimore County 123999, Baltimore, Maryland, USA; Queens College Department of Biology, Queens, New York, USA

**Keywords:** *Streptomyces*, heavy metal tolerance, bacteriophages, bacteriophage genetics, phage immunity

## Abstract

Bacteriophages HazuAndZazu and Tubberson, belonging to the BI1 and BC1 subclusters, are Caudoviricetes with a siphoviral morphology that infect *Streptomyces* species. They have GC contents of 59.5% and 71.5%, and genomes 55,823 and 39,028 bp long, respectively. Annotation of cluster BC phage Tubberson includes an immunity mechanism.

## ANNOUNCEMENT

The sequencing of bacteriophages, viruses that infect bacteria (1), holds significant medical importance in the development of phage therapy, a treatment targeting pathogenic bacteria (2). Phage therapy reduces harm to organisms targeted by bacteria and has fewer side effects than antibiotic treatments due to their high specificity (1), allowing phages to evade bacterial defenses through their concurrent evolution (3). In this study, bacteriophages HazuAndZazu and Tubberson were isolated using the bacterial host *Streptomyces mirabilis*.

**Fig 1:**
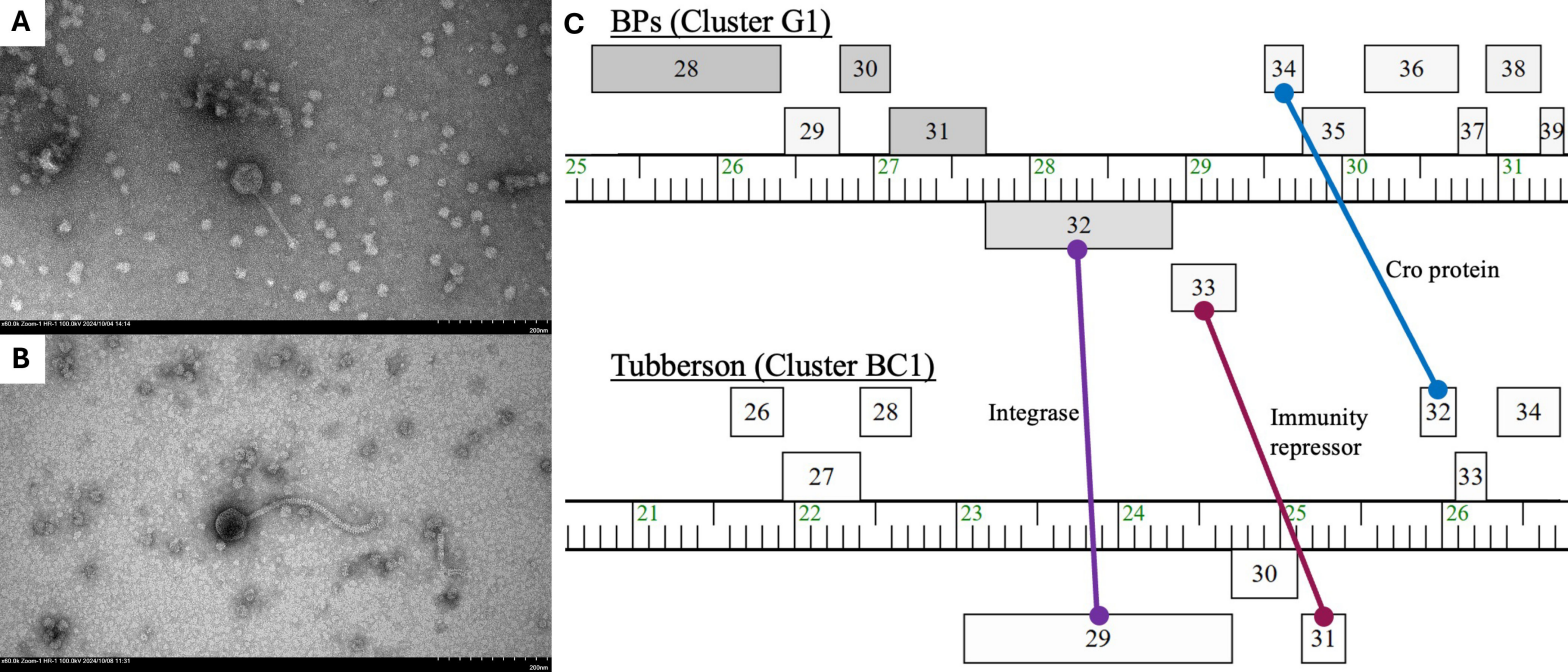
Characterizations of *Streptomyces* phages Tubberson and HazuAndZazu. (**A**) TEM image of Tubberson with a 200 nm scale bar. Fresh lysate was negatively stained with 2% uranyl acetate and imaged using a Hitachi HT7800 120 kV TEM with an AMT Nanosprint15 B digital camera. Phage particle measurements are shown in Table 1. (**B**) TEM image of HazuAndZazu with a scale bar of 200 nm. Fresh lysate was negatively stained with 2% uranyl acetate and imaged using a Hitachi HT7800 120 kV TEM with an AMT Nanosprint15 B digital camera. Phage particle measurements are shown in Table 1. (**C**) Feature map of Tubberson, a BC1, compared with BPs (accession no. EU568876), a G1, displaying conserved synteny for a known integration-dependent immunity system. Ruler markings correspond to 1,000 bp, and genes are represented with numbered boxes above and below rulers, corresponding to rightward and leftward transcription mechanisms. A serine integrase (feature 29) in phage Tubberson corresponds to a tyrosine integrase on BPs (feature 32). An immunity repressor and subsequent Cro protein of Tubberson (features 31, 32) corresponds to features 33 and 34 of BPs, respectively. Tubberson features 31 and 32 contain HHPred alignments for N-terminal DNA-binding domains specific to repressors. This, combined with syntenic alignment of an integrase, supports the presence of this immunity repressor mechanism in cluster BC.

*Streptomyces* phages HazuAndZazu and Tubberson were isolated from soil samples in Catonsville, MD, and Burtonsville, MD, respectively (Table 1). They were isolated following procedures outlined in the SEA-PHAGES *Phage Discovery Guide* (4). In short, collected samples were treated with phage buffer (10 mM Tris (pH 7.5), 10 mM MgSO_4_, 68 mM NaCl, and 1 mM CaCl_2_) and plated on nutrient agar plates (BD Difco, containing 10 mM MgCl_2_, 8 mM Ca(NO_3_)_2_, and 0.5% glucose) after combining with *S. mirabilis* and tryptic soy top agar (BD) (4). Samples were incubated for 24 and 48 h at 30°C and underwent at least three rounds of plaque purification, after which fresh lysates were produced from plates with near confluent lysis. Uranyl acetate-stained transmission electron microscopy (TEM) revealed siphoviral morphologies for both Tubberson (Fig. 1A) and HazuAndZazu (Fig. 1B).

**TABLE 1:** Characterizing the genomic contents of *Streptomyces* bacteriophages HazuAndZazu and Tubberson

Parameter	HazuAndZazu	Tubberson
Sequencing		
Sequencing instrument	Illumina	Illumina
Library prep kit	NEB ultra II library kit	NEB ultra II library kit
Number of reads	1,949,963	2,103,460
Length of reads (bp)	100 bp single-end reads	100 bp single-end reads
Shotgun coverage (x)	3,431	5,195
Phage characteristics		
Genome length (bp)	55,823	39,028
Genome character	Linear	Circular
Genome end type	3′ sticky overhang	Circularly permuted
GC content	59.50%	71.50%
Cluster assignment	BI, subcluster BI1	BC, subcluster BC1
Sample characteristics		
Collection date	7 September 2024	4 September 2024
Collection location coordinates	39.25772 N, 76.70801 W	39.10035 N, 76.9289 W
Isolation temperature	30°C	30°C
Capsid length (nm)	58	56
Capsid width (nm)	58	57
Tail length (nm)	246	99
Genome characteristics		
Total number of identified genes	82	53
Number of genes with identified functions	24	33
Number of genes without identified functions	58	20
Number of orphams	1	0
Number of tRNA genes	0	0

DNA was isolated using the Promega Wizard DNA clean-up kit from freshly harvested plate lysates and sequenced by the Pittsburgh Bacteriophage Institute. Sequencing of HazuAndZazu and Tubberson was performed with the Illumina MiSeq sequencing platform using the NEB Ultra II Library Kit and 100 bp single-end reads (Table 1). Raw sequencing reads had a shotgun coverage of 3,431× and 5,195×, respectively. Consed v29 and Newbler v2.9 were used for assembly and determination of genome ends as described (5–7)

Genome lengths and end characteristics can be found in Table 1. HazuAndZazu and Tubberson were assigned to clusters BI1 and BC1, respectively, in the Actinobacteriophage Database (PhagesDB) as described (8, 9). Genome annotations were completed using PECAAN v20241104 (10) and DNA Master v5.23.6 (11) embedded with Glimmer v3.02b (12) and GeneMark v2.5p (13). ARAGORN v1.1 (14) and tRNAscanSE v2.0 (15) were used to call tRNAs (Table 1). Positional annotations were investigated according to the SEA-PHAGES guidelines (16, 17) using the Starterator database v583 (18) and BLASTp v2.16.1+ (19) to manually assess conserved start sites. NCBI BLASTp v2.16.1+ (19), HHPred (probability >90%) (20), and Phamerator v583 (21) embedded with DeepTMHMM v1.0.42 (22) were used to assess functions. Default settings were used.

Features 29, 31, and 32 of cluster BC phage Tubberson correspond to a previously characterized immunity repressor mechanism also found in clusters G, I, N, and P. These clusters consist of leftward transcription of an integrase and an immunity repressor, then rightward transcription of a Cro (control of repressor’s operator) protein (23). Syntenic alignment combined with HHPred domain alignment of the N-terminal DNA-binding domains of specified features indicates that this mechanism is present in cluster BC phages as well (Fig. 1C).

## Data Availability

HazuAndZazu is available in GenBank with accession no. PV105562 and Sequence Read Archive (SRA) no. SRX27501635. Tubberson is available in GenBank with accession no. PV105563 and Sequence Read Archive (SRA) no. SRX27501639.
